# A review of 640 Oral squamous cell carcinoma cases in Nigeria

**DOI:** 10.4317/jced.53680

**Published:** 2017-06-01

**Authors:** Ahmed-Oluwatoyin Lawal, Akinyele-Olumuyiwa Adisa, Olajumoke-Ajibola Effiom

**Affiliations:** 1 FMCDS. Senior Lecturer/Consultant, Department of Oral Pathology, College of Medicine, University of Ibadan, Nigeria; 2 FMCDS. Senior Lecturer/Consultant, Department of Oral and Maxillofacial Pathology & Biology, College of Medicine, University of Lagos, Nigeria

## Abstract

**Background:**

Oral squamous cell carcinoma (OSCC) is the most prevalent malignant neoplasm in the oral cavity and accounts for 70% to 90% of all oral malignant neoplasms. The aim of this study was to examine the demographic distribution of OSCC in five Tertiary Health centres in Nigeria.

**Material and Methods:**

All cases diagnosed as OSCC during the period from 1970 -2014 were retrieved from the records of five teaching hospitals in Nigeria. Hematoxylin and eosin stained histological slides of all cases that had a diagnosis of OSCC were reviewed for confirmation and inclusion. Data from all the centers was collated at the University College Hospital, Ibadan by 2 researchers. The data was entered into and analyzed with the SPSS for Windows (version 20.0; SPSS Inc. Chicago, IL). Simple descriptive and comparative analyses were done, with the test of statistical significance set at *p* ≤ 0.05.

**Results:**

A total of 640 cases of OSCC were seen out of 1560 oral malignant neoplasms representing 41% of all the oral malignancies seen. The mean age of occurence of OSCC was 55.5 (±17.0) years and a peak age incidence in the sixth and seventh decades of life. OSCC occurred more in males (60.9%) than females (39.1%) with a male: female ratio of 1.6:1. The well differentiated OSCC with 309 (48.3%) cases, was the most common grade, while the moderately differentiated and poorly differentiated OSCC accounted for 232 (36.2%) and 92 (14.4%) cases respectively.

**Conclusions:**

This study showed that OSCC is more common in males, most commonly seen in the 60-69 age group and the commonest site of occurrence was the mandibular mucosa. The well differentiated OSCC was the most common histology sub-type.

** Key words:**Oral squamous cell carcinoma, tongue, palate, mandible.

## Introduction

According to World Health Organization, carcinoma of oral cavity in males in developing countries, is the sixth commonest cancer after lung, prostrate, colorectal, stomach and bladder cancer, while in females, it is the tenth commonest site of cancer after breast, colorectal, lung, stomach, uterus, cervix, ovary, bladder and liver (1). In the oral cavity, oral squamous cell carcinoma (OSCC) is the most prevalent malignant neoplasm and has been reported to account for 70% to 90% of all oral malignant neoplasms (2,3). There is global variation in the incidence of OSCC with the Indian sub-continent presenting with particularly high incidence and prevalence, presumably, because of the predominant habits of chewing tobacco, betel quid and areca-nut (4).

OSCC occurs most often in males who are above 40 years of age (5,6). Some authors have reported that up to 98% occurs above 40 years of age, with the incidence rising from an overall average of 3-4 cases per 100,000 per annum at all ages, to 100 cases per 100,000 per annum in those over 75 years of age (7). However, early exposure to tobacco use in western countries and habitual abuse of smokeless tobacco by teenagers in the Indian subcontinent has caused an increase incidence of OSCC amongst younger people (7,8). More so, the increasing incidence of OSCC in young people and especially in those who do not smoke or use alcohol has been associated with increased infection by Human Papilloma Virus (HPV) occasioned by changing sexual preference of young people (9).

The aim of this study was to analyse the demographic distribution of OSCC in five tertiary Health facilities in Nigeria.

## Material and Methods

All cases diagnosed as OSCC during the period from 1970 -2014 were retrieved from the records of the University Teaching Hospitals of Lagos, Ile-Ife, Ibadan, Port Harcourt, and Zaria. Hematoxylin and eosin stained histological slides of all cases that had a diagnosis of OSCC were reviewed for confirmation and inclusion. Researchers in each center retrieved records to obtain age, gender and location. The lesions were classified into; well differentiated, moderately differentiated and poorly differentiated according to broder’s classification. Included parameters were documented according to a proforma designed by the researchers. Data from all the centers was collated at the University College Hospital, Ibadan by 2 researchers. The data was entered into and analyzed with the SPSS software version 20. Simple descriptive and comparative analyses were done, with the test of statistical significance set at *p* ≤ 0.05. Ethical approval for the study was obtained from the UI/UCH Ethical Review Committee.

## Results

A total of 640 cases of OSCC were seen out of 1560 oral malignant neoplasms representing 41% of all the oral malignacies seen at the 5 centres. OSCC occured with an age range of 4-108 years with a mean age of 55.5 (±17.0) years and a peak age incidence in the sixth and seventh decades of life both accounting for 43.0% of total number of cases seen.  Table 1 shows age and gender distribution of OSCC from the five centres. OSCC was seen more in males (60.9%) than females (39.1%) with a male:female ratio of 1.6:1. The mean age for males was 54.8±16.4 whilst that for females was 56.7±17.7 but there was no statistically significant difference in the mean ages between males and females (*p*=0.24).  Table 2 shows the site distribution of OSCC; the mandible with 169 (26.4%) cases was the most common site of occurrence followed by the maxillary gingiva with 109 (17.0%) cases. The palate recorded 92 (14.4%) cases whilst 59 (9.2%) and 27 (4.2%) cases ocurred in the tongue and floor mouth respectively.

**Table 1:**
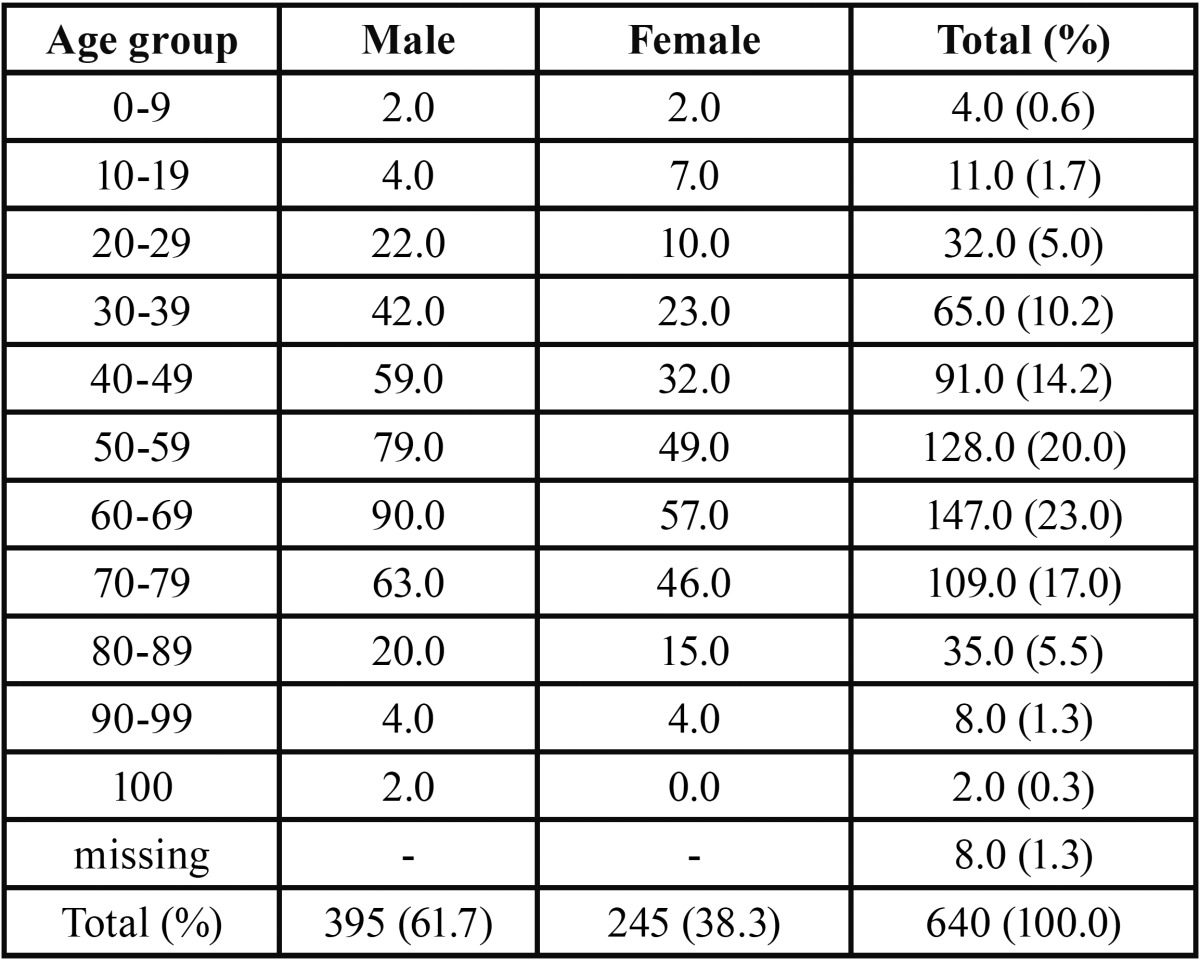
Age and gender distribution of OSCC cases.

**Table 2:**
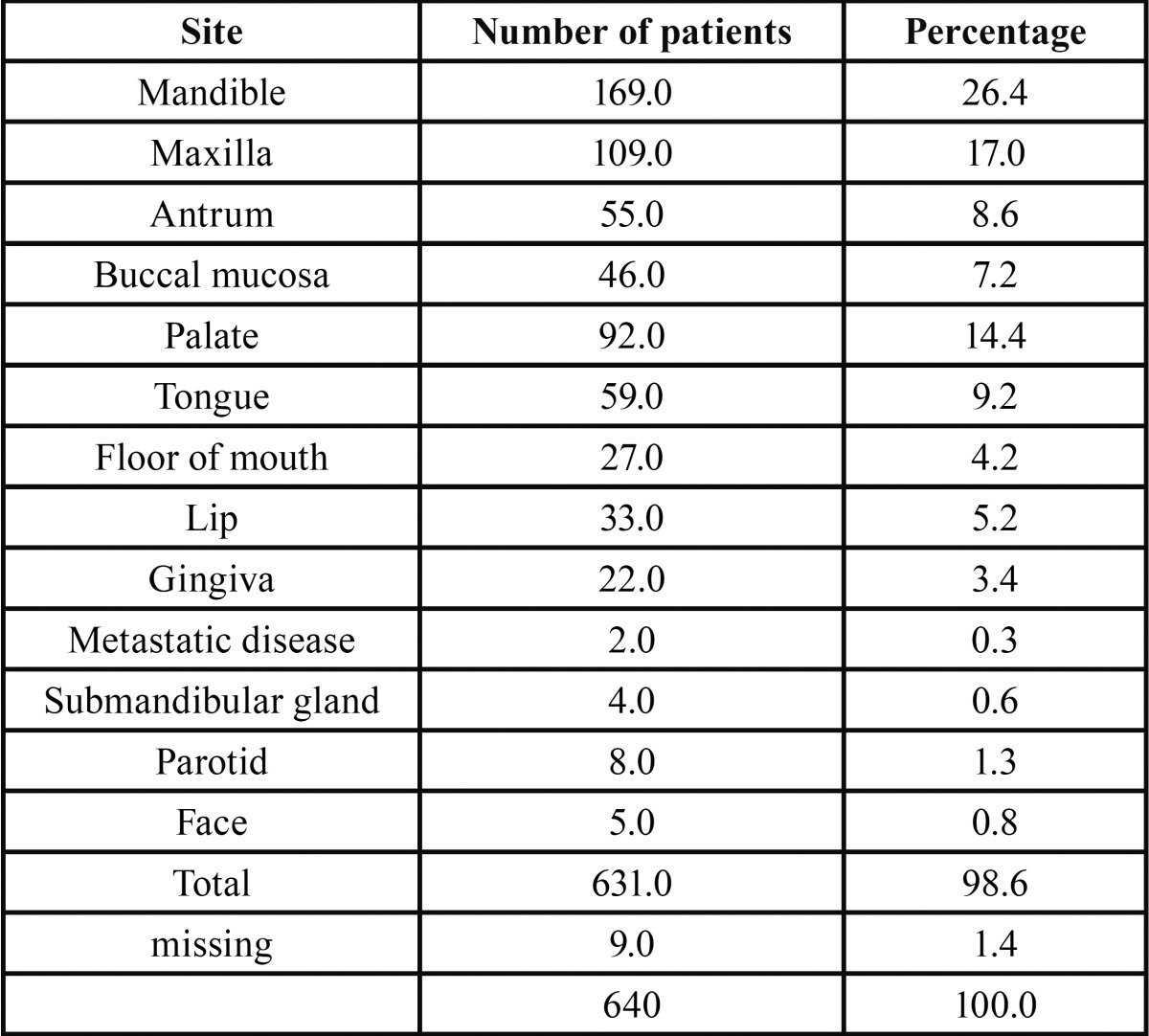
Site distribution of OSCC.

Concerning Histologic grade, well differentiated OSCC with 309 (48.3%) cases, was the most common grade, while the moderately differentiated and poorly differentiated OSCC accounted for 232 (36.2%) and 92 (14.4%) cases respectively ( Table 3). The well differentiated OSCC occurred at peak age of 60-69 and had a mean age of 55.6±16.8 while the moderately differentiated OSCC occurred at peak age of 60-69 had a mean age of 56.7±16.43. In the same vein, poorly differentiated OSCC had mean age of presentation of 51.7±18.2 and had a peak age incidence of 40-49. However, there was no statistically significant difference in age between histological grades of OSCC (*p*=0.43, using one way anova). Although, OSCC occurred more in males in all 3 Histologic grades, the poorly differentiated OSCC had a much higher male: female ratio of 2.8:1, compared with 1.4:1 and 1.3:1 for well differentiated OSCC and moderately differentiated respectively.

**Table 3:**
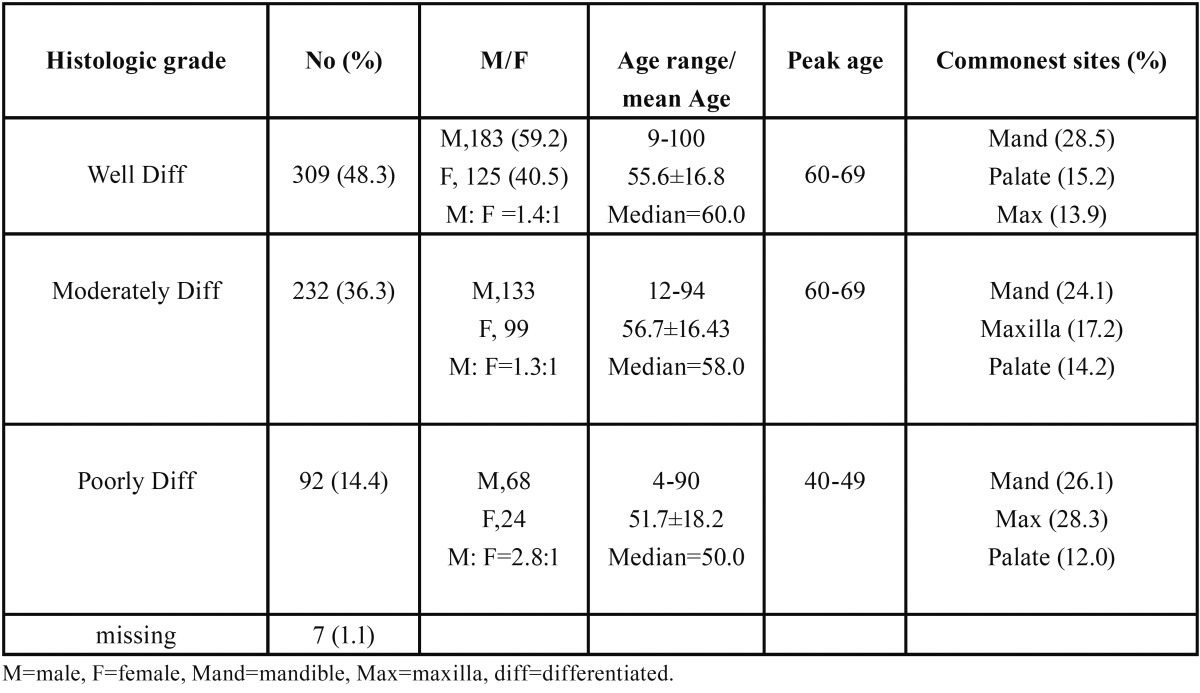
Sex, age and site distribution according to histologic grade.

## Discussion

Oral malignant neoplasms constitute a significant global health problem with reports signifying that they are the sixth most common malignancies worldwide and, together with malignancy of the pharynx, are the third most common malignancies in the developing world (10). The finding of this study that OSCC constitutes 41% of all oral malignancies was in sharp contrast to most previous studies which showed that OSCC accounts for 70-95% of all oral malignancies (3,11-13). However, our finding was consistent with that of Ajayi *et al.* (8) who reported that OSCC accounts for 44% of all oral malignancies. The reason for the wide disparity in the relative frequency of OSCC in this study compared to most other studies is not immediately apparent, but subtle difference in aetiology and predisposing factors are suggested.

The tongue is generally regarded as the commonest site of occurrence for OSCC. Santos *et al.* (14) suggested that consumption of alcohol from an early age may account for up to 40% of OSCC in the tongue as reported in their study in Brazil. Choi *et al.* (15) from Korea, Dimba *et al.* (16) from Kenya and Iamaroon *et al.* (17) from Thailand also reported 20.4%, 20.1% and 42.8% occu-rrence of OSCC in the tongue respectively. However, results from this present study found that the mandibular mucosa with 26.4% of cases was the most common site while only 9.2% occurred in the tongue which was in agreement with studies by Chidzonga *et al.* (3) and Effiom *et al.* (18) who reported highest incidence in the mandibular gingiva with values of 21.1% and 31.8% in mandibular gingiva respectively. The reason for the relatively high rate of occurrence of OSCC in the mandibular mucosa, the floor of mouth and tongue has been related to the fact that carcinogens in tobacco, alcohol, or foods dissolve in saliva and tend to pool in gravity dependent areas of the mouth (18). The reason for the disparities in the occurrence of OSCC in the mandibular mucosa, the tongue and floor of mouth from different studies, however, needs to be further investigated. The Lip is an uncommon site for OSCC in blacks (18) because of the protective effect of melanin and this is confirmed in this study with only 3.6% OSCC occurring in the Lip. Santos *et al.* (14) reported a 31.6% occurrence in the lower lip and thought that this might be due to the high exposure of their patients to sunlight radiation as the region of study was in close proximity to the equatorial line.

In the present study, the finding of a peak age incidence for OSCC in the 60-69 age group was in consonance with reports that OSCC occurred in older patients. Also, only 17.3% of cases were seen below 40 years of age. This seems to confirms the long-held view that OSCC is seen more in older people as reported in several previous studies (19-22). On the contrary, a study by Effiom *et al.* in Lagos found a peak age incidence in the 40-49 age group and 40% occurrence of OSCC below the age of 40 years.

The well differentiated OSCC with 48.3% of cases was the most common histology grade while the poorly differentiate grade with 14.4% was the least common in this study. This was in agreement with previous studies that found well differentiated OSCC to be the most common histologic grade and the poorly differentiated grade to be the least (3). On the contrary, Effiom *et al.* found the poorly differentiate grade (47.6%) to be the commonest and the moderately differentiated (19.7%) as the least common.

This multicentre study showed that OSCC is more common in males, most commonly seen in the 60-69 age group and the commonest site of occurrence was the mandibular mucosa. The well differentiated OSCC was the most common histology sub-type. However, the differences between some of our findings and some previous studies from Nigeria, such as peak age of occurrence and the most common histologic sub-type, need further investigation.
